# Community-based management induces rapid recovery of a high-value tropical freshwater fishery

**DOI:** 10.1038/srep34745

**Published:** 2016-10-12

**Authors:** João Vitor Campos-Silva, Carlos A. Peres

**Affiliations:** 1 Departamento de Ecologia, Centro de Biociências, Universidade Federal do Rio Grande do Norte, Natal, RN 59072-970, Brazil; 2 Centre for Ecology, Evolution and Conservation, School of Environmental Sciences, University of East Anglia, Norwich NR4 7TJ, UK

## Abstract

Tropical wetlands are highly threatened socio-ecological systems, where local communities rely heavily on aquatic animal protein, such as fish, to meet food security. Here, we quantify how a ‘win-win’ community-based resource management program induced stock recovery of the world’s largest scaled freshwater fish (*Arapaima gigas*), providing both food and income. We analyzed stock assessment data over eight years and examined the effects of protected areas, community-based management, and landscape and limnological variables across 83 oxbow lakes monitored along a ~500-km section of the Juruá River of Western Brazilian Amazonia. Patterns of community management explained 71.8% of the variation in arapaima population sizes. Annual population counts showed that protected lakes on average contained 304.8 (±332.5) arapaimas, compared to only 9.2 (±9.8) in open-access lakes. Protected lakes have become analogous to a high-interest savings account, ensuring an average annual revenue of US$10,601 per community and US$1046.6 per household, greatly improving socioeconomic welfare. Arapaima management is a superb window of opportunity in harmonizing the co-delivery of sustainable resource management and poverty alleviation. We show that arapaima management deserves greater attention from policy makers across Amazonian countries, and highlight the need to include local stakeholders in conservation planning of Amazonian floodplains.

Although freshwater ecosystems comprise only 0.8% of Earth’s surface^1^, they host one third of all vertebrate species worldwide^2^, and have always played a critical role in societal development throughout human history. Currently, freshwater environments and wetlands are top global scale conservation priorities, because they are rapidly becoming the most threatened ecosystems, particularly in the tropics, with rates of species loss substantially higher than those of terrestrial environments^3^.

Many of these overexploited and increasingly degraded freshwater environments can be described as socio-ecological systems (SES), where social norms, ecological relationships and biophysical interactions are dynamic, complex and reciprocal^4^. Human settlements, for example, are heavily dependent on freshwater resources such as fish, and the top-down structure of entire fish communities is often governed by the intensity of human overexploitation^5^. Conservation and management of fish stocks are therefore essential to the economic stability and social wellbeing of floodplain dwellers. Conservation of freshwater ecosystems is widely considered an intractable problem affected at different spatial scales, and is inextricably related to the often extolled but rarely reconciled major challenges for humanity in the new millennium: biodiversity conservation, improved quality of life, and poverty alleviation^6^.

Establishing the framework for sustainable resource use in locally co-managed SESs is often a herculean task. This challenge is particularly difficult partly because resource populations are affected by both biotic and abiotic factors, in addition to the landscape dynamics of human exploitation pressure. For example, marine fisheries respond to large-scale spatial patterns, such as latitude, elevation, annual precipitation, and ecosystem primary productivity^7^. At smaller scales, several other important variables can govern freshwater resource availability including landscape and habitat heterogeneity^8^, water chemistry, and plankton productivity^9^.

Setting aside and implementing well-managed protected areas is but one way of achieving sustainable resource use^10^. For example, effectively established marine protected areas have resulted in significant seascape-scale increases in fishery yields^11^. Moreover, there are contentious discussions that remain unresolved about the role of sustainable-use protected areas in realistically reducing poverty and promoting other social benefits, mainly in developing countries^12^. The rationale behind the thorny issue of reconciling biodiversity conservation with local socioeconomic needs can be mainly justified at two levels. First, implementation and maintenance of existing protected areas, particularly large tropical reserves, are rarely effective due to scarce financial and human resources, and inherent surveillance difficulties in enforcing reserve regulations against a myriad of increasing external threats^13^. Therefore, formal alliances with reserve residents can decentralize resource management, strengthen full-time surveillance systems, and reduce overall conservation costs^14^. Secondly, protected areas can enhance income opportunities, creating direct social and economic benefits for local people^15^. For both of these reasons, legally occupied sustainable-use reserves now exceed people-free strictly-protected reserves in terms of both numbers and total area throughout the tropics^16^.

There are good examples of local communities that have been effectively empowered to manage their own resources. These approaches are often referred to as Community-Based Management (hereafter, CBM), whereby local people with a vested interest in sustainable-use activities undergo an empowering process to gain management control of their local natural resources^17^. This has been independently demonstrated to work in different resource management systems, for example to strengthen the sustainability of coral reef fisheries^18^, convert commercial hunters into conservationists in Afrotropical forests^19^ and improve conservation outcomes in Himalayan forests^14^. However, well-grounded examples of positive ecological outcomes from CBM schemes have been rarely demonstrated^20^.

A rare noteworthy example of community empowerment of artisanal fisheries management has been occurring in lowland Amazonia^21^. With growing market demand and technological innovation, large-scale commercial fishing pressure on Amazonian fish stocks has been escalating since the early 1960s^22^. This fueled the emergence of community-based management initiatives, whereby fisherfolk began to restrict access by large commercial fishing boats into lakes near their communities^23^. These initiatives, whenever they can be formalized, have been variously referred to as ‘Fishing Accords’ between subsistence and commercial fishing interests and have had a strong effect on local fisheries management. In 1993, government agencies legally sanctioned these local agreements as a formal fisheries management tool, which has since become a powerful strategy to prevent overexploitation of important fish species^24^. Since 1999, such fishing accords, based on a strong social organization movement, paved the way to the development of a promising community-based management system focused on the exploitation of arapaima or *pirarucú (Arapaima gigas*, Arapaimidae), a target species of marked importance in Amazonian history and prehistory.

*Arapaima spp.* represents an apex predator in Amazonian fish communities and Earth’s largest scaled freshwater fish, reaching >3.0 m and >220 kg (Supplementary Information, Fig. S1). The alpha taxonomy of this monotypic genus is poorly understood, and some new Amazonian species may yet be described^25^. Adult arapaima exhibit high levels of parental care, protecting its fry at all times, which contributes to its relatively low fecundity^26^. Fry-guarding adults produce a mucous secretion, which flows through the water creating a safety net that maintains a more cohesive family unit, and reducing natural predation on young^27^. Due to its high ecological, economic and cultural value, large body size, late maturity, and small brood sizes, Arapaima *spp.* is highly vulnerable to overexploitation, and has been driven to local extinction at many localities^28^. Surprisingly, however, *A. gigas* is currently listed as ‘data deficient’ in the most recent IUCN Red List of Threatened Species.

Here, we provide a quantitative assessment of how CBM can promote the recovery and conservation of arapaima, one of the most important tropical freshwater fisheries, while generating both significant income and economic food security for local livelihoods across Amazonian floodplains. We examine the effects of different spatial scales of protection (protected lakes within and outside protected areas), community management regime, distance to nearest markets, and limnological and landscape-scale variables associated with 83 monitored lakes spread across a ~600-km section of the Juruá River of western Brazilian Amazonia. We further identify the patterns of local perception on arapaima population growth as witnessed across 41 semi-subsistence communities surveyed over the last 10 years. Finally, we discuss CBM initiatives as a powerful tool for Amazonian floodplain conservation in decentralizing responsible decision-making over natural resources, while serving the interests of both biodiversity conservation and local livelihoods.

## Methods

### Study Landscape and Social Context

**Figure 1:**
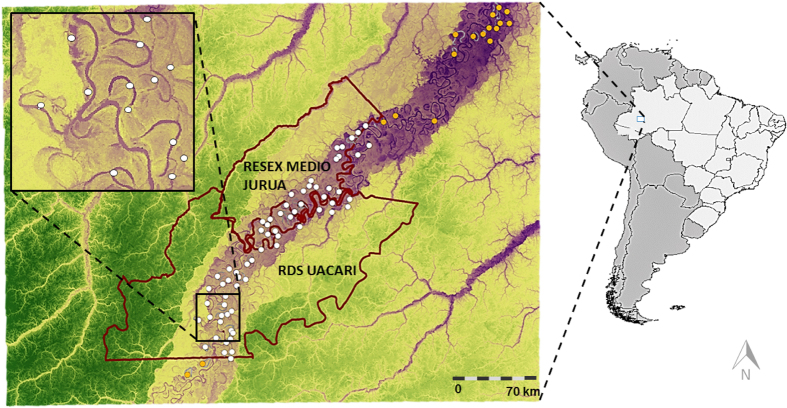
Distribution of 87 floodplain lakes sampled across a ~600-km segment of the Juruá River of western Brazilian Amazonia. White and orange circles indicate lakes inside and outside protected areas, respectively. Dark-red lines show the boundaries of two contiguous sustainable-use forest reserves, which amount to a combined area of 886,176 ha. This map was generated in ArcGIS 10.3 ( http://www.esri.com).

This study was carried out at 80 floodplain lakes inside and outside (upriver and downriver) of two large contiguous sustainable-use reserves along the middle section of the Juruá River, the second-largest white-water tributary of the Amazon River (Fig. 1). The ~14,000-km^2^ study landscape contains two main forest types: 17.7% of seasonally-flooded (*várzea*) forest along the wide floodplain and 82.3% of upland (*terra firme*) forest which is rarely if ever inundated^29^. The wet and dry seasons coincide with periods of high (January–June) and low floodplain water levels (August–November), with a prolonged flood pulse often exceeding 10 m in amplitude^30^.

We note the unusually high level of socio-political organization of the local communities occupying this region. Over much of the last century, natural latex exploitation by rubber tappers was the dominant economic activity in central-western Brazilian Amazonia. However, as government subsidies dwindled, rubber extractivists gradually succumbed to extreme rural poverty. This created a serious need for social self-organization fueling local demands for sustainable-use forest reserves, where traditional extractive lifestyles were granted communal territory rights, thereby preventing more predatory forms of land use^31^.

In this context, the federally-managed 253,227-hectare Médio Juruá Extractive Reserve (RESEX Médio Juruá) was created in 1997. Located on the west bank of the river (5°33′54″S, 67°42′47″W; Fig. 1), this reserve is legally occupied by some 2000 people distributed across 23 villages. This was followed by the creation of the state-managed 632,949-hectare Uacari Sustainable Development Reserve (RDS de Uacari) (5°43′58″S, 67°46′53″W) where ~1200 people live in 32 villages. In addition to those 45 local communities, we also monitored 14 communities outside these protected areas. The local economy is sustained by fisheries, slash-and-burn cassava agriculture, and non-timber forest products such as oil seeds and palm fruits.

### ‘Fishing Accords’

To ensure food and economic security for rural communities, *Fishing Accords* in the mid Juruá region were negotiated between local communities at the two focal reserves, communities outside those reserves, and the Fishermen Cooperative of Carauari, the nearest town. However, this is the first attempt to evaluate the effectiveness of these fishing agreements.

**Figure 2:**
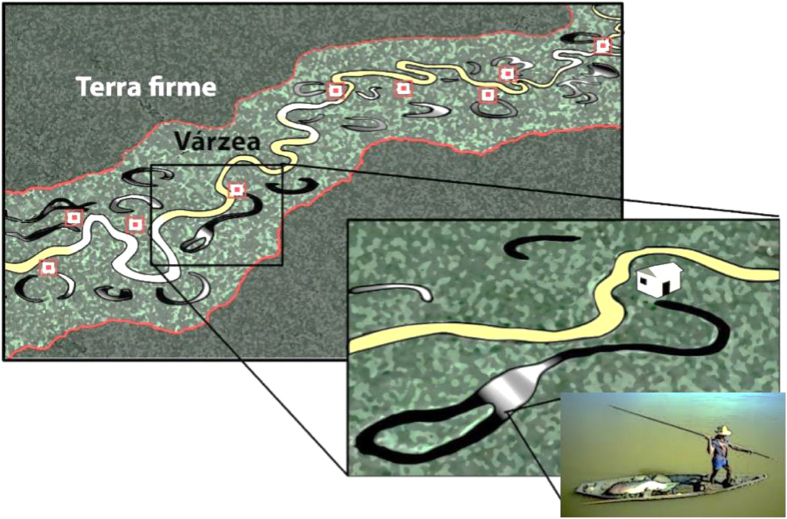
Section of the Rio Juruá floodplain showing the upland (terra firme) forest (dark green area) and floodplain várzea forest (light green area), containing oxbow lakes and levees. Community-based full-time surveillance scheme protecting lakes during the dry season is made possible by a wooden floating house placed at a strategic access point at the mouth of the lake (red squares). Different families in each resident community, who are often armed with a shotgun, take turns guarding the lake against underhand poachers. Intermediate inset figure shows details of a protected lake; small inset figure shows a harpoon fisherman in a dugout canoe harvesting arapaimas.

These agreements established three categories of lake resource access during the dry season, when lakes become discrete geographic features in the landscape: (1) **Open-access lakes** contain free-for-all resource pools and remain available for any fishing interests, including commercial fishing boats; (2) **Subsistence-use lakes** are designed to supply local subsistence needs and restrict access to only subsistence artisanal fishermen from the resident community responsible for guarding that lake; and (3) **Protected lakes** are managed by local communities primarily as stock recovery and arapaima management sites, and exclude both commercial and subsistence fishing boats. A floating wooden guard post is usually erected at the main strategic entrance of the lake, thereby serving as a full-time armed vigilance unit managed by the resident community (Fig. 2). During the arapaima management season, some of protected lakes are harvested by the resident community for only a brief dry-season period of up to 5 days per year, according to a previously set proportional harvest quota based on the number of adult and juvenile arapaima censused at that lake in the previous year.

Annual arapaima counts along the mid Juruá started at several lakes in 2005, whereas lake management was implemented in 2010 by a partnership between local communities, local associations and government agencies. Arapaima counts take place during the low-water season at each monitored lake every year, and the census data are forwarded to IBAMA (Brazilian Natural Resources Agency), which then authorizes a lake-specific harvest quota of up to 30% of all adults (>1.5 m in length) counted, depending on the fish processing requirements of the resident community and other extenuating factors.

### Arapaima counts

*Arapaima spp.* is an air-breathing fish that is highly adapted to hypoxic and anoxic environments^27^, thereby frequently coming to the surface to breathe air, which facilitates direct sightings and counts (supplementary video S1). This census method is highly effective, was developed and repeatedly field-tested in Central Amazonian floodplains, with the specific objective of quantitatively surveying arapaima populations (see ref. 32 for further details). This census method produces population size estimates that are strongly correlated with those from mark-recapture abundance estimates^32^. Along the Juruá, this technique involved the collaborative participation of up to 20 previously trained and highly experienced arapaima fishermen per lake, who could detect air-breathing arapaima on the lake surface through both visual and acoustic cues. During systematic censuses of each lake, each fisherman working collectively sequentially covered a non-overlapping lake area ranging from 0.2 to 2.0 ha, depending on local constraints such as macrophyte coverage and lake area, to avoid double-counts.

During census periods, each observer remained silent on the lake margins and counted all arapaima detected within each census area over multiple 20-min periods (coinciding with the mean observed air-breathing interval at which they become visible), which were synchronized across observers. These counts could distinguish two main size classes: juveniles (1.0–1.5 m in length) and adults (>1.5 m in length). In very large lakes, counts were conducted over more than one census session often taking the whole day, until the entire census had been completed. To preclude any detectability problems due to background noise, arapaima counts were restricted to favourable weather conditions, which excluded rainy days and strong winds.

### Arapaima revenues

For each lake and each family household, we estimated the total revenue derived from sales of legally harvested arapaima for local communities of the Juruá region. This was based on ~6200 adult arapaima counted in 2015 at 26 protected lakes. In doing so, we assumed (i) the maximum legally permitted offtake of 30% of the recently censused adult population (Sept–Oct 2015) at managed lakes; (ii) the average dressed weight of butchered and clean carcasses ready to be commercialized (71.3 kg per adult); (iii) the mean market price in the nearest local town (R$ 5.5 ≈ US$2.08 per kg), which is a conservative estimate of market value but more realistically reflects transaction prices actually paid to floodplain dwellers; and (iv) a mean monetary exchange rate of US$1 = R$2.64 (December 2014). We include both subsistence and open-access lakes in these calculations for comparison, but in practice, arapaima catches from these lakes can be consumed locally or bartered, but cannot be sold to external traders.

### Datasets and Variables

To understand the determinants of arapaima population sizes within oxbow lakes, we examined systematic arapaima census data obtained at 83 lakes located along a ~500-km fluvial distance along the Rio Juruá (31 protected; 34 subsistence; and 18 open-access lakes; Fig. 1) that had been surveyed at least once during the dry season of 2013. We also had access to yearly dry-season arapaima count data (2005–2015) from most of these lakes, obtained by a collaborative institutional partnership, which yielded a total of 269 counts at 77 lakes (mean = 3.49 annual counts per lake).

For the full set of 83 lakes (Dataset 1), we obtained explanatory data on fisheries management history and a range of landscape variables extracted for each lake using ArcGIS (version 10.2). Predictors of arapaima stock sizes across those lakes included: *Protection area status*: if the lake was inside or outside any protected area; *Lake management category*: open-access, subsistence, or protected; *Lake area:* including both open-water and macrophyte cover; *Distance to nearest community*: the true nonlinear path distance on foot or boat used by local users, which was measured using a GPS; *Distance to nearest market:* expressed as the nonlinear fluvial travel distance from the lake entrance to the town of Carauari port; *Distance to the river channel*: the Euclidean distance between the lake entrance and the main Juruá river channel; *Connectivity:* presence of a perennial levee or secondary channel connecting the lake to any larger water body; and *Water geochemistry*: ‘black’ or ‘white’, defined as the locally perceived amount of suspended alluvial sediments in the lake water column.

Secondly, for a subset of 43 of the 83 lakes (Dataset 2), we quantified proxies of primary productivity of the lakes and obtained detailed limnological data based on both field and laboratory measurements of water samples collected during both the dry (low-water) and wet (high-water) seasons. These included: *Depth:* maximum lake depth; *Water transparency:* estimated using a Secchi disk; *Conductivity:* measured in μS/cm using a conductivity meter; *Macrophyte cover:* initially mapped in the field and then independently measured using 5-m resolution RapidEye^©^ images, which we purchased for the entire study area; *Phytoplancton biomass:* estimated based on both dry- and wet season water samples and chlorophyll-a measurements using high-performance liquid chromatography (HPLC); and *Total phosphorus and nitrogen*: determined using light absorbance at 882 nm. A more detailed description of these variables, measurements and hypotheses are presented in Supplementary Table S1.

### Local perception surveys

We conducted 63 semi-structured interviews at 41 local extractive communities containing at least six households. A total of 26 and 15 of these communities were located inside and outside our two focal protected areas, respectively. Interviewees were selected if they were heads of households who were both highly experienced arapaima fishermen and had been continuous full-time residents at any given community for >10 years. During these interviews, we objectively asked about the overall perception of the local arapaima population status (i.e. increasing, decreasing, or stable) in terms of the perceived size of the present population within one or more lakes that had been frequently visited by local villagers during the dry season, against perceived background population trends over the last 10 years. The experienced fishermen have been chosen according the leadership indication, at least one per community.

We also conducted 28 interviews with self-declared formerly illegal arapaima fishers at 13 communities, 12 of which inside the reserves and one outside. These experienced fishers had since abandoned illegal fishing practices and are currently working with the arapaima management program. They also reported on perceived socio-economic changes since the onset of the management program. Essentially, we asked about major perceived changes in local livelihoods after the implementation of CBM. We also assessed the level of importance of any given response, in terms of its overall relative frequency across all interviews. Each interview lasted up to 15 min, and was facilitated by the overall experience of resident fishermen in terms of frequent observational exposure to arapaima populations at community lakes, and fishing effort over at least a decade using harpoons, gillnets, or both.

In this study, we adhere to the full set of legislative and ethical specifications to conduct the research within or outside Brazilian protected areas, including the way we handled local interviews and conducted arapaima surveys. Our methods were explicitly carried out in accordance with the formally approved legal guidelines and licensing requirements as stipulated by the Brazilian Ministry of Science and the Environment (SISBIO license number 45054). We can confirm that all sampling protocols were approved by Brazilian law; and that any data acquisition activities that may have involved people or third parties were conducted with their explicit and clear-headed consent, once they had been completely informed by native Portuguese speakers about the nature and objectives of the research. We further declare no conflicts of interest in reporting the results of this research work.

### Data Analysis

To understand the local environmental and management determinants of arapaima population size, we examined Datasets 1 and 2, using the number of adult and juvenile arapaima estimated from systematic counts at each focal floodplain lake as response variables. Dataset 3 was then used to examine the variation in population size and annualized population growth rates from multiple counts within each lake.

First, we ran generalized linear models (GLMs) to examine the variation in recent (2013) counts within the full set of 83 lakes (dataset 1) as a function of all potential predictors. Second, we performed GLMs to examine the variation in arapaima population size within the subset of 43 lakes for which limnological data, including proxies of productivity, were available (dataset 2). Our patch metrics, lake management, and limnological fixed effects are listed above for these datasets. Although arapaima population sizes should scale to lake area, we opted to retain this variable as a fixed effect, rather than as an offset measure, because both census detectability and habitat suitability within lakes were likely highly variable. However, because ecological studies using count data are often affected by overdispersion, a quasi-poisson and negative binomial distribution are often used^33^. We used the latter because the variance-mean relationship provided a better fit.

Third, we used generalized linear mixed models (GLMMs) and a negative binomial error structure to examine variation in all 269 yearly arapaima counts (2005–2015) considering the same set of predictors, but nesting population counts within the 77 lakes surveyed at least twice (range = 2–8 yearly counts), with lake identity defined as a random factor. Fourth, we examine the variation in annualized population growth rates (*G*_*N*_) within and across lakes by calculating percentage changes in population sizes between any two consecutive dry-season counts (including both adults and juveniles) within the same lake 
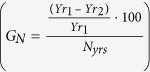
, which did not necessarily take place in consecutive years. This yielded 186 positive or negative G_*N*_ estimates (% yr^−1^) across 71 lakes exploited by 26 local communities.

We first selected the most parsimonious random intercept structure by identifying the model with the lowest Akaike Information Criterion corrected for small sample size (AICc) with all fixed effects added^34^. ΔAICc is calculated as the difference between the AICc of each model and the lowest AICc, with ΔAICc <2 interpreted as substantial support that the model belongs to the set of best models. Akaike weights give the probability that a model is the best model, given the data and the set of candidate models^35^. Models were fit with lmer in the *lme4* package and every model combination examined with the *MuMIn* package^36^ within the R platform (R Development Core Team 2015). When comparing models that varied in their random effects but not fixed effects, models were fit using restricted maximum likelihood (REML). Finally, we calculated the hierarchical partitioning of each explanatory variable.

Because arapaimas exhibit seasonal movements during the flood pulse (JVCS and CAP, unpubl. data), it is possible that population sizes could be homogenized through source-sink dynamics across lakes near one another regardless of their prevailing local management history. We therefore examined the spatial structure of the data across all 83 lakes (yielding 3,160 pairwise Euclidian distances between any two lakes; mean = 73.6 ± 55.6 km; range = 1.3–223.3 km) to investigate the degree to which lakes could be considered as spatially independent. However, there were no differences in model fits between whether or not we included the geospatial structure of the data (expressed as the x, y centroids of lake localities) as additional terms in spatial autoregressive models explaining arapaima stock sizes within lakes based on key environmental and management predictors (χ^2^ likelihood ratio test, *P* = 0.15). In addition, stock sizes in open-access lakes were unrelated to physical distances to the nearest protected lake (R^2^ < 0.001, *P* = 0.966). We therefore decided to interpret, rather than formally incorporate, large-scale spatial effects in any subsequent analyses.

## Results

### Population responses to community management

**Figure 3:**
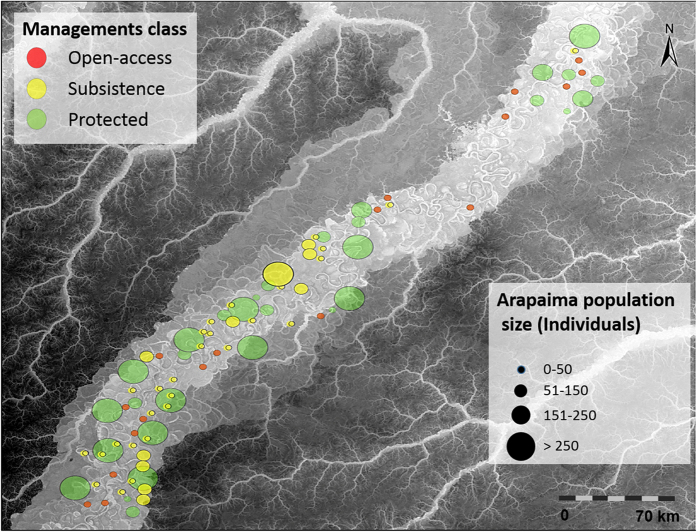
Spatial distribution of protected (green circles), subsistence (yellow circles) and open-access lakes (red circles) along a ~500-km section of the Rio Juruá, Western Brazilian Amazon. Symbol sizes are scaled according to the 2013 arapaima annual population counts. Background elevation map of the study region shows a colour gradient from higher (dark grey) to lower terrain (light grey), with várzea floodplains and oxbow lakes shown in very light grey. This map was generated in ArcGIS 10.3 ( http://www.esri.com).

**Figure 4:**
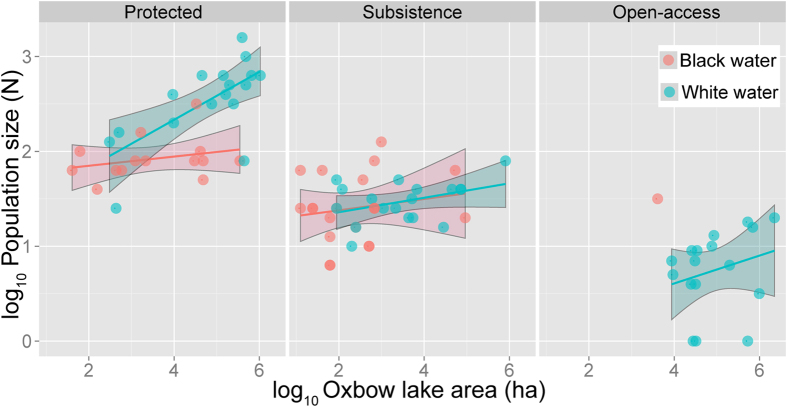
Arapaima population size as a function of floodplain lake area for protected, subsistence and open-access lakes. These relationships were broken down into two main categories of lake water geochemistry, black- and white-water lakes. Intercepts (but not slopes) are significantly different across lake management classes. Slopes are significantly different between black- and white-water protected lakes, with population sizes expanding with lake area much faster in the latter.

There was a dramatic positive response of local arapaima populations to community-based lake management regime (Figs 3 and 4). Lake protection status, as enforced by local communities, explained 71.2% of the variation in population sizes in dataset 1 and 66.8% in dataset 2. Population size estimates were significantly different between the three lake management classes (p < 0.001), and averaged over three orders of magnitude from 304.8 (±332.46, *N* = 31) individuals in protected lakes to 34.1 (±24.4, *N* = 34) and 9.2 (±9.8, *N* = 18) individuals in subsistence and open-access lakes, respectively (Supplementary Fig. S2). This becomes even more striking considering that open-access lakes were much larger (222.5 ± 172.1 ha, *N* = 18) than protected lakes (126.5 ± 117.4 ha, *N* = 31), resulting in a mean arapaima population density 131-fold higher in the latter (open-access lakes: 0.002 ind. ha^−1^; protected lakes: 0.294 ind. ha^−1^). Whether or not a lake was formally within or outside protected areas was not a significant predictor of arapaima stock sizes (Supplementary Fig. S2).

Lake management class also induced marked differences in the relative abundance of both adults and juveniles (Anova, F_75,2_ = 14.6, P < 0.001). In protected lakes, there were no numerical differences between these age classes, with adults representing an average ratio of 53.4% (±3.7%) of all individuals (162.9 ± 170.3 adults vs. 141.9 ± 171.6 juveniles). In contrast, subsistence lakes were proportionally dominated by juveniles (71.6 ± 3.3%), with mean counts of 9.4 (±10.7) adults and 24.7 (±19.23) juveniles (Supplementary Fig. S3), almost certainly because the persistent year-round harvesting in those lakes selectively targeted adults, whereas live captures of juveniles (<1.5 m in length) were always released.

Positive growth rates were widespread in arapaima populations in both protected and subsistence lakes following the onset of community-based management (Supplementary Fig. S4). Annualized population growth rates across lakes of different management categories (subjected to at least two annual counts) were negative for seven open-access lakes (median = −7.1%, *N* = 21 G_*N*_ estimates), but invariably positive for all 41 subsistence (22.7%, N = 125) and 29 protected lakes (34.6%, N = 123). In exceptional cases, arapaima stocks from one year to the next grew five to eight-fold in subsistence lakes, and five to 16-fold in protected lakes. Comparing the first and last years of management at each lake, population sizes increased by 213.3% in protected lakes (mean interval = 5.4 ± 2.5 yrs, *N* = 29) and 193.5% in subsistence lakes (3.9 ± 2.0 yrs, *N* = 41), but declined or remained at persistently low numbers at open-access lakes. For example, the very small Year_1_ populations recorded at open-access lakes (range 0–24 ind.), which were surveyed over three consecutive years (2013–2015), declined even further or remained unchanged (median change = −11.1%), except for two lakes (Lago Santo Antônio and Lago Baliera) whose initially small stocks more than trebled, perhaps showing some signs of source-sink demographic subsidies from neighbouring protected and subsistence lakes.

### Local management, landscape and limnological effects

**Figure 5:**
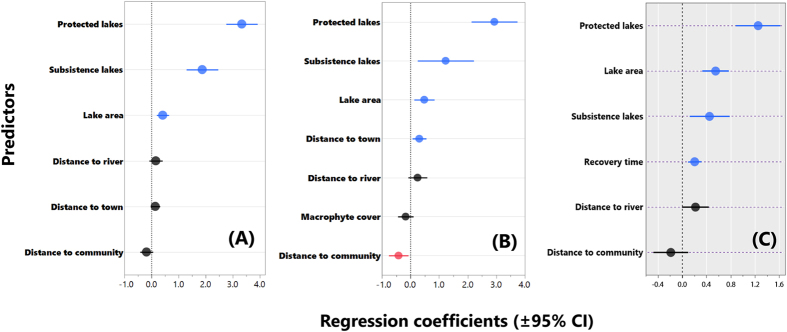
Coefficient estimates (±95% confidence intervals) showing the magnitude and direction of effects of different local and landscape scale variables on arapaima stock sizes and population growth rates within floodplain lakes of Western Brazilian Amazonia. Arapaima stock sizes were modelled with (**A**) generalized linear models (GLMs) using a set of 83 lakes censused in the dry-season of 2013; (**B**) GLMs for a subset of 43 lakes where detailed limnological and lake productivity variables were also quantified; and (**C**) generalized linear mixed models (GLMMs) for a subset of 77 lakes for which 269 annual counts (2005–2015) were available. ‘Time’ in panel C interaction terms refers to recovery time or the number of years since the onset of sustainable management at any given lake. Explanatory variables were standardized prior to analyses.

Whether we considered (i) all 83 lakes where at least a single arapaima count was available for the same dry season (2013), (ii) the 77 lakes subjected to at least two annual counts, or (iii) the smaller subset of 43 lakes for which limnological data were available, community-enforced mode of lake access always explained the most amount of variance in stock sizes and population growth rates (Fig. 5A–C), with protected lakes always containing the largest or fastest growing populations, followed by subsistence lakes. As expected, although lake area was a significant positive predictor of stock sizes, its effect was consistently smaller than that of lake management class. Recovery time (years) was a significant positive predictor of stock sizes for those lakes counted over more than one year, with high growth rates for protected lakes showing no evidence of slowing down (Supplementary Fig. S4). Nonlinear walking distance to the nearest local community had a significant negative effect on population growth rates (Fig. 5B and Supplementary Fig. S5), presumably because protection measures were inherently more effective at lakes in close proximity to a village, provided residents remained vigilant to enforce widely agreed community rules in light of Fishing Accords. None of the limnological variables were important predictors of population descriptors, except for the proportion of macrophyte cover which was negatively correlated with stock sizes (r = −0.41). Further model details can be seen in Supplementary Table S2).

### Local perception of stock recovery

**Figure 6:**
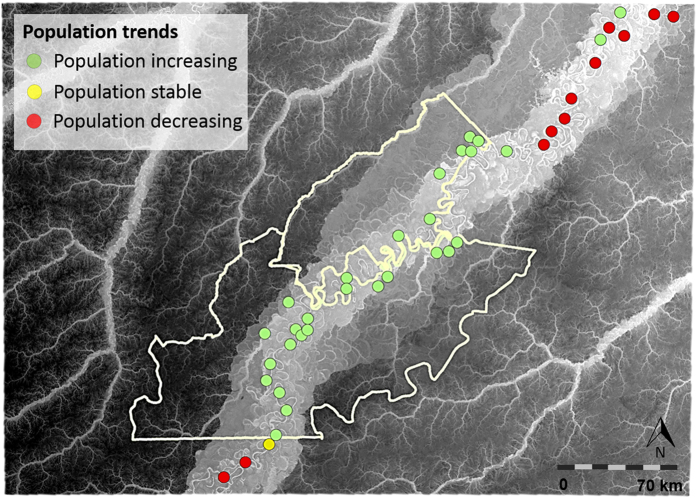
Local perceptions on arapaima population trajectories based on semi-structured interviews with experienced arapaima fishermen. Red and green circles indicate communities (and community lakes) for which local informants reported either a decline or an increase in arapaima population sizes over the last decade, respectively. Yellow circles indicate stable populations that have not appreciably changed over time. Yellow lines represent the boundaries of the two contiguous sustainable-use forest reserves, which may or may not contain lakes referred to during interviews. This map was generated in ArcGIS 10.3 ( http://www.esri.com).

Perception surveys with experienced fishermen confirmed the increasingly evident notion that local arapaima populations have been growing inside but not outside protected areas. However, at two communities well outside the protected areas, there was unanimous consensus that arapaima stocks were also increasing since a community management scheme was established (Fig. 6). This again lends support to the idea that lake management in itself overrides the wider effects of protected area status in determining arapaima population trajectories. Socioeconomic benefits accrued from population recovery, as listed by formerly illegal fishermen, were in order of importance: generation of local income, strengthening of cultural values, growing “pride” in the community, and a more equitable distribution of profits from fisheries (Supplementary Table S3).

### Arapaima revenues

**Figure 7:**
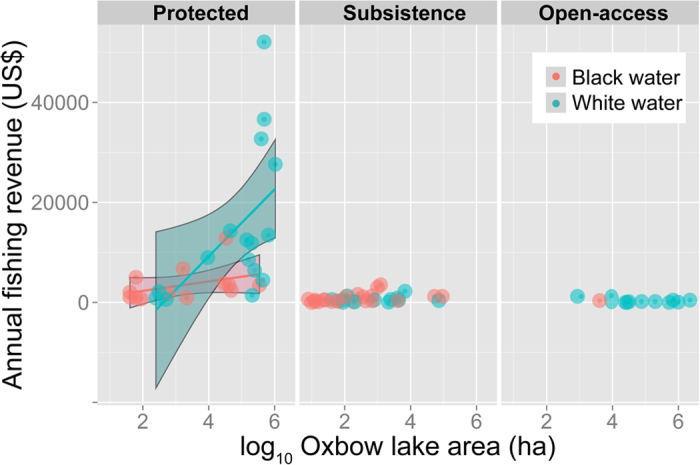
Gross fishing revenues per local community across three classes of lake resource protection through community-based management, which were further broken down into two main lake types in terms of water geochemistry. Slopes for protected lakes are significantly different between black- and white-water lakes, with arapaima populations in increasingly larger lakes accruing much higher revenues.

The bulk of local income benefits accrued from fisheries management was restricted to protected lakes, with subsistence and open-access lakes flat-lined at virtually zero commercial value (Fig. 7). Protected lakes could derive total fishing revenues from arapaima stocks averaging US$10,601 [95% CI: US$5,393, US$15,808] every year, provided that full compliance with management rules takes place and total allowable catches (TACs) are harvested. However, some exceptionally large white-water lakes could yield as much as US$52,093 yr^−1^ if the officially sanctioned TAC had been sold. This translates into mean annual revenues per community household of US$1,046.6 [95% CI, US$497, US$1,596], considering the 14.4 ± 8.5 families per community (range = 4–30) that were engaged in arapaima management activities. These family revenues were positively correlated with the number of years since the onset of the CBM program (r = 0.791, *P *< 0.001). Arapaima population growth (and potential revenues) scaled strongly to lake area in white-water lakes, but not in black-water lakes, which appear to be intrinsically less productive in terms of carrying capacity and stock recovery (Fig. 7). White-water lakes on average yielded significantly higher mean revenues (US$1,662.2 ± 350.6) than black-water lakes (US$449.4 ± 395.2; *P* = 0.025).

Finally, we used a predictive model to estimate the average recovery time to achieve a stock size of 1000 adult arapaimas per lake, which could generate a reasonable annual income (≈US$44,491) for the resident community managing that lake. We used as predictors the size of the lake, the distances to the nearest local community and to the main river channel, and the nearest market town. This shows that achieving a stock size of 1000 individuals would take a mean recovery time of only 7.5 to 8.0 years from the onset of the CBM program for white-water and black-water lakes, respectively, and at most 14 years considering the upper 95% CI of our estimates (Supplementary Fig. S6).

## Discussion

Even some of the most severely underfunded protected areas can be powerful instruments of tropical biodiversity conservation^37^. However, most tropical protected areas are designed on the basis of known terrestrial ecology guidelines, even if they contain extensive freshwater ecosystems. In Amazonia, the overwhelming majority of protected areas are designed to protect forest biodiversity, whereas increasingly threatened freshwater biotas remain highly neglected^38^. This emphasizes the need to rethink how best to protect freshwater ecosystems, but this is hindered by severe political resistance to create new, or expand existing, protected areas^39^. In fact, protected area policy will likely succumb to serious setbacks in many tropical countries, where existing reserves are being downgraded, downsized or degazetted^40^. Given this unfavourable scenario and government suspension of spawning season closures^41^, decentralization of conservation policies and alliances with highly engaged local stakeholders can become powerful tools^14^.

Recovery of arapaima fisheries has been suggested within some Amazonian protected areas^21^,^42^. Although our study reinforces these findings, we further show that CBM initiatives, in which floodplain lakes are the management unit of interest, can impart even stronger positive outcomes for conservation, even if implemented outside the boundaries of formally protected areas. Local stewardship, direct *in situ* surveillance, full-time presence, and management of high-value fish stocks were the most important factors in boosting arapaima population sizes across a wide range of lakes. Proximity to the nearest community was a key proxy of effective protection, largely because community management rules and *de facto* exclusion of competing resource users cannot be easily enforced in more remote lakes. Moreover, arapaima represents an umbrella species in floodplain lakes, so that protecting their stocks brings about collateral benefits to other important taxonomic groups, such as freshwater turtles^43^ and caimans (JVCS and CAP, unpubl. data).

Although arapaima populations in subsistence lakes are not fully protected, these lakes are in theory closed to commercial fishing boats. This resulted in a much reduced and more size-selective offtake, with live catches of smaller individuals released back into the lakes. This explains both the intermediate arapaima population size compared to either open-access or protected lakes, and the high number of juveniles. In fact, subsistence lakes likely serve a critical role in juvenile recruitment, and ensure largely exclusive access to highly-selective artisanal fisheries targeting large populations of smaller-bodied detritivore and frugivore fish, which provide the mainstay of animal protein for Amazonian floodplain dwellers^21^.

Although protected area context has been shown to be imperative to ensure arapaima stock recovery elsewhere^21^,^42^, and remains an important overarching scale of protection, we argue that the Juruá sustainable-use reserves were not only important in protecting harvest-sensitive stocks, but also provided more favourable conditions for successful CBM establishment. Community-level socio-political organization and local compliance are critical determinants of successful resource management^44^, and these community traits were conspicuously missing in villages outside protected areas.

Beyond the protected area and CMB effects on arapaima populations, understanding the environmental factors that govern resource distribution and abundance is critical to the establishment of resource use guidelines^45^. First, all other things being equal, larger lakes should be expected to contain larger arapaima populations, and should be prioritized for management (cf. 45). Distances to the nearest town and to the main river channel could also important predictors of stock sizes. The mid Juruá town of Carauari serves as a convergence point for an operational fleet of over 800 variable-sized fishing boats, which largely supply chilled fish to a few wholesale middlemen who monopolize the regional fish trade. Therefore, harvesting pressure on fish stocks is substantially higher near the town than at distant sites up- or down-river, for which travel costs are higher, as well documented in other CBM schemes^46^. The floodplain distance effect can be explained simply in terms of physical access, because more remote (and often older) floodplain lakes far from the meandering main channel are rarely visited by commercial fishing boats.

Considering the subset of 43 lakes where detailed limnological variables were quantified, macrophyte cover was an important yet ambiguous predictor of arapaima population size. The habitat heterogeneity created by macrophytes positively affects aquatic species richness^47^ — providing shelter, refuge and foraging sites for many species^48^— but hinders physical access to gillnet and harpoon fishermen. The macrophyte zone also provides a critical food supply for juvenile arapaima^49^, so we expected the coverage of macrophytes and number of arapaima to covary positively. Surprisingly, however, our data show an inverse relationship, possibly due to a sampling artifact. High levels of macrophyte cover impair counts of arapaima whose detectability is associated with surfacing in open-water^32^, thereby super-inflating sampling imprecision. In this study, arapaima population sizes were most likely underestimated in lakes dominated by large areas of macrophytes.

Water geochemistry also played important roles in arapaima population size as proxies of primary productivity. Although this effect size was weak, more productive white-water lakes with high phytoplankton biomass likely supported larger numbers of arapaima. In sum, arapaima populations within dry-season lakes responded strongly to both human exploitation and some biophysical variables. Therefore, we can identify both top-down and bottom-up factors affecting populations of an Amazonian harvest-sensitive large-bodied fish, so that large white-water lakes, particularly those near local communities should be prioritized for local co-management initiatives both *within* and *outside* protected areas.

Arapaima population growth trajectories in protected lakes were a function of time. This is consistent with the results from Mamirauá Sustainable Development Reserve, Brazil, where the arapaima population increased nine-fold in eight years^21^. We cannot yet estimate the maximum carrying capacity of these lakes, but all protected subpopulations were still growing, partly fueled by the annual input of alluvial nutrients brought in by the rising floodwaters, which likely enhances the resilience of exploited animal populations^48^. An example worth highlighting is the Marari Grande lake (Supplementary Table S4), which had been entirely unprotected and available for professional fisheries until 2008. Experienced fishers from the nearest community reported that this lake had long been depleted, and that arapaimas had been locally extirpated. After only 7 years of protection this lake yielded the largest arapaima population in our study landscape (~2,020 individuals). Like elsewhere in Amazonia^22^,^24^, much of this depletion process was driven by less selective large-scale commercial fishing boats, which come from far afield to harvest arapaima and other high-value fish from unprotected lakes. The rapid reversal in this situation resulted from a local initiative to guard the Marari Grande and other protected lakes, which suddenly enforced the exclusion of professional fishing boats from those lakes.

Throughout our study landscape, experienced fisherfolk have reported that arapaima stocks gradually dwindled to very low numbers during the heyday of commercial fishing boats of the 1980s, to the point of perceived local extinction from most lakes in the region. However, this predicament was only reversed with the onset of negotiated fishing closures in both protected and subsistence lakes. This becomes clear when we assessed stock sizes in open-access lakes, which have been bearing the brunt of overfishing. These free-for-all lakes clearly fall under a ‘tragedy of the commons’ scenario^50^, whereby scramble competition for valuable fish resources accelerate overexploitation. During the first years of exploiting an open-access lake, the benefits are disproportionately concentrated on the few commercial fishermen harvesting that lake, to the detriment of all local users. Over time, the pressure on harvest-sensitive resources became prohibitively high, so the population eventually collapsed. Fishing Accords are thus a concrete example of positive communal organization, defining boundaries, and establishing rules of governance that are instrumental in managing common-pool resources, thereby precluding resource collapse^51^. Moreover, such local initiatives can trigger rapid co-benefits in multi-species population recovery in previously depleted lakes, thus increasing the overall fisheries productivity of both those lakes and the neighbouring waterscape^22^.

Although arapaima population growth is restricted to managed lakes, the map resulting from local perception surveys indicates that the recovery is widespread throughout the reserves (Fig. 6), whereas arapaima stocks are perceived to have bottomed out outside protected areas. This clear perception pattern is largely due to the absence of widely known protected lakes outside protected areas. However, in the few cases where grassroots management plans were self-established by communities outside protected areas, the arapaima population has been clearly showing an early growth trajectory. This can be seen at Lago Grande (Supplementary Table S4), a 294-ha lake outside the protected areas where the arapaima population increased from ~30 to over 1200 individuals in only 3 years of CBM lake protection.

Yet the degree to which population growth is due to internal recruitment or immigration remains poorly known. For example, it is inconceivable that a low-fecundity fish population can increase 3900% in only 3 years, such as in Lago Grande. Rapid stock recovery must therefore be due to both local reproduction and lateral migration during the high-water season^52^, whereby protected lakes likely function as demographic sources. This is consistent with the long-range movements exhibited by arapaima during the flood pulse. For example, one of the juveniles we tracked at RDS Uacari using VHF radio-telemetry moved ~30 km in a few days (JVCS and CAP, unpubl. data). A better understanding of seasonal subpopulation movements during floodwaters is critical in designing a landscape-scale network of protected, semi-protected and unprotected lakes that maximizes fisheries productivity for all regional stakeholders.

Integrated development strategies that render biodiversity conservation truly compatible with poverty alleviation are still too rhetorical but often elusive^53^. Locally co-managed arapaima fisheries in Amazonian floodplains could become an important window of opportunity because this can generate income for thousands of families, apparently without significant wider opportunity costs, for example, in depressing fish catches for excluded commercial stakeholders. Indeed, we show that protected lakes have recently become something analogous to a high-interest savings account, ensuring an average value of nearly US$10,600, assuming the maximum allowable harvest quota of up to 30% of adults and an adequate level of communal organization and compliance. This rare cash-earning opportunity has also improved the socioeconomic welfare of local communities, enhancing education and health services. Moreover, resident communities managing protected lakes can count on an unprecedented annual windfall payment every year, which enables often prohibitive private or communal investments, such as house refurbishment and purchase of expensive equipment. In addition to local income and social welfare, emergency funds can be generated from arapaima sales, which have covered costs of urgent travel and medical care at nearby urban centers or the state capital (Manaus) in case of serious illness or accidents. This form of immediate access to health care, often resulting in life-saving interventions, is unavailable from state health services. In sum, we show that arapaima CBM schemes can become a colossal welfare enhancement opportunity at relatively low costs. For example, covering all additional expenses for both counting the arapaima population at a large lake (>120 ha) each year, and enforcing anti-poaching vigilance at that lake costs only US$700 per year.

Socioeconomic benefits listed in our perception surveys with formerly illegal arapaima fishermen include local income, more equitable distribution of fisheries profits, and maintenance of cultural integrity. Prior to the management program, illegal arapaima sales were distributed throughout the annual calendar, rather than accrued as a lump sum following the collective dry-season offtake. This cash windfall contributes further administrative benefits in terms of village and household scale financial organization, enabling local managers to invest, for example, in community infrastructure. Fisheries CBM has also been instrumental in propagating traditional knowledge. Children often help during the fish offtake season, while simultaneously learning about arapaima ecology and capture techniques. Experienced fishermen also reported that CBM communities now show a stronger “sense of pride” flowing from the management program, which has been reinforced by positive media coverage disseminating this success story. All of these direct and collateral benefits have greatly strengthened local empowerment, providing positive feed-back on the emergence of the management program as a whole^54^. Finally, local income distribution within villages has become significantly more egalitarian following arapaima CBM because everyone can participate, whereas illegal catches were previously oligopolized by a few highly skilled fishermen.

In recent decades, the Brazilian government, local organizations, local communities, and conservation NGOs have attempted to develop participatory management strategies with a broad base of local uptake^55^. One of the most important features of arapaima CBM in this study is the robust social organization of both small and large communities, which ensures the dialogue, articulation and partnership among stakeholders at different institutional scales, ensuring that the lake protection system operates properly. The management structure is essentially communitarian, whereby the resident community coupled with other lake users define the rules of engagement and use of that environment, so that the spatial patterns of resource access are defined at both individual and collective levels. Moreover, CBM becomes more robust with the adhesion of additional partners because of higher financial benefits, stronger self-monitoring of management effectiveness, and greater collective vigilance in terms of formal or informal law compliance. We also note the strong participation of grassroots institutions, including reserve resident cooperatives and associations of rural producers, which were erected by community members themselves. This ensures that decision-making is in fact in the hands of local communities that were hitherto disenfranchised and had no political voice. This further empowers local resource-users to co-adapt and fine-tune management guidelines according to local culture, which is critical to the success of conservation interventions^56^.

However, the long-term viability of Arapaima CBM will depend on a number of externalities. Legal trade of sustainably harvested fish requires producers to meet a number of sanitary preconditions stipulated by Brazilian health authorities. This demands minimum processing equipment and infrastructure, which most local communities do not yet have. These historically deprived communities have so far been isolated from public policies, so meeting even minimum certification standards will clearly require catalysis from government subsidies.

But perhaps the most important incidental challenge to be considered is the likely urban market saturation from many competing arapaima management initiatives at several Amazonian river basins, which would considerably increase supply but depress market prices. Moreover, illegal fishing also remains a significant competitive threat^57^, because offtakes from unknown sources are delivered to consumers in large amounts at substantially lower cost. A third wildcard threat to sustainable arapaima management comes from aquaculture in that many Amazonian fish farms are now beginning to produce captive-bred arapaima for local and regional markets^58^. Understanding these market bottlenecks will be vital for the continuity of successful community-based wild fish management. In doing so, government agencies, NGOs and other stakeholders should consider the nuances of trade chains to enhance market conditions for novice CBM traders. Managing wild arapaima populations remains a highly promising conservation opportunity, but maintaining economically viable CBM in the long term will critically depend on solving these challenges.

### Arapaima, a paradigm “fish of change”

Local Fishing Accords are not new, as they have been established at several Amazonian sites for nearly four decades, although the outcomes have been elusive^59^. However, arapaima fisheries have profoundly changed the concept of local fishing agreements. This fishery and its associated value reinforces the justification of protecting lakes, and allocating much community time and endeavor to this end.

Undoubtedly, arapaima management is a rare window of opportunity to harmonize the often incompatible goals of sustainable resource management and poverty alleviation. All similar efforts across the Amazon are also showing positive results^21^,^42^. This is significant because these local communities rarely have any alternative cash-earning opportunities. Arapaima management can thus positively empower local communities, and fishing agreements can be instrumental in the sustainable management of aquatic resources in tropical floodplains, thereby serving as an excellent stratagem to recruit allies with full-time physical presence in protecting these threatened environments. Yet, there is not enough federal and state government investment in Brazil—in terms of information transfer, technical input and trade subsidies—to catalyze the initiation and consolidate similar CBM programs despite widespread popular demand. Finally, we emphasize that fishing agreements alone are not a panacea and cannot substitute the creation of large protected areas, because these also ensure the continuity of many complex ecological processes that sustain baseline resource productivity^60^.

## Additional Information

**How to cite this article**: Campos-Silva, J. V. and Peres, C. A. Community-based management induces rapid recovery of a high-value tropical freshwater fishery. *Sci. Rep.* **6**, 34745; doi: 10.1038/srep34745 (2016).

## Supplementary Material

Supplementary Information

Supplementary Video S1
