# Supplementary material for: Community-based management induces rapid recovery of a high-value tropical freshwater fishery
Source: Sci Rep. 2016 Oct 12;6:34745. doi: 10.1038/srep34745 (PMC5059620; doi:10.1038/srep34745)
Supplement: Supplementary Information [file srep34745-s1.pdf]

Supporting Information

Community-based management induces rapid recovery of a high-value tropical freshwater fishery

João Vitor Campos-Silva and Carlos A. Peres

1. Supplementary Information Tables

Table S1. Description of explanatory variables, details of measurements and hypotheses examined in this study.

Variable	Description	Variable type	Dataset	Hypotheses
**Protection status**	If the lake is located within or outside protected areas	Binary	1 and 2	Lakes inside protected area hold larger arapaima population sizes
**Management class**	Class of community-based management: Protected, Subsistence or Open-access	Categorical	1 and 2	The arapaima population is largest in protected lake, followed by subsistence lakes, and smallest in open-access lakes
**Lake area**	Total area (ha)	Continuous, extracted using ArcGIS 10	1,2 and 3	Larger lakes hold larger arapaima populations
**Distance to nearest community**	Distance on foot travelled by local users	Continuous, extracted in ArcGIS 10	1 and 2	Lakes near the community have smaller arapaima populations, due to high fishing pressure
**Distance to market**	Fluvial (nonlinear) distance to the nearest market town	Continuous, extracted using ArcGIS 10	1 and 2	Lakes near large markets have smaller arapaima populations, due to high fishing pressure
**Distance to river channel**	Nonlinear distance along water bodies and the Jurua river channel	Continuous, extracted using ArcGIS 10	1 and 2	Lakes near the river have smaller arapaima populations because they are more accessible
**Connectivity**	Presence of a perennial streams connecting the lake to another water body	Continuous, extracted in ArcGIS 10	1 and 2	Highly connected lakes have larger arapaima populations, likely due to immigration
**Lake water type**	Lakes with a primary hydrological connection to either the main river or	Categorical	1	White-water lakes have larger arapaima populations than

	streams draining upland catchments are defined as “white-water” or “black-water”, respectively.			black-water lakes, due to higher productivity and nutrient load
**Depth**	Maximum depth	Continuous, measured in situ	2	Deep lakes have larger arapaima populations
**Transparency**	Estimated with a Secchi disk	Continuous, measured in situ	2	Lakes with higher transparency have larger arapaima populations, due to higher planktonic productivity
**Macrophyte cover**	Mapped in the field and then measured again using RapidEye© images purchased for the entire study area	Continuous, measured both in the field and extracted using ArcGis 10	2	Lakes with high macrophyte cover have larger arapaima populations
**Chlorophyll-a**	Chlorophyll-a was estimated using the high performance liquid chromatography (HPLC) method	HLPC method in the laboratory	2	Lakes with higher levels of chlorophyll-a hold larger arapaima populations

Table S2. Human perception of ongoing socioeconomic changes following the implementation of Arapaima population management within lakes guarded by any given local community.

Dataset 1				
**Model**	Predictors	AICc	Δ AICc	Weight
**1**	*Management class + Area + Distance to town*	833.3	0.00	0.163
**2**	*Management class + Area*	834	0.67	0.117
**3**	*Management class + Area + Distance to community + Distance to river*	831.2	0.89	0.105
**4**	*Management class + Area + Distance to community*	834.3	1	0.099
**5**	*Management class + Area + Distance to community + Distance to town*	834.6	1.26	0.087
**6**	*Management class + Area + Distance to community + Distance to river + Distance to town*	835.1	1.73	0.069
**7**	*Management class + Area + Distance to river + Distance to town*	835.2	1.86	0.064
Dataset 2				
**1**	*Management class + Area + Distance to community + Distance to town*	452.5	0.00	0.091
**2**	*Management class + Area + Distance to community + Distance to town + Distance to river*	452.8	0.35	0.076
**3**	*Management class + Area + Distance to community + Distance to town + Macrophyte coverage*	453.3	0.87	0.059
**4**	*Management class + Area + Distance to town*	454.0	1.5	0.043
**5**	*Management class + Area + Distance to community + Distance to town + Depth*	454.2	1.71	0.039
**6**	*Management class + Area + Distance to town + transparency*	454.3	1.83	0.036
**7**	*Management class + Area + Distance to community + Distance to town + Transparency*	454.4	1.95	0.034
**Dataset 3**				
**1**	*Management class + Recovery time + Lake area + Distance to river + Distance to community*	637.62	0.00	0.265
**2**	*Management class + Lake area + Recovery time + Distance to river + Management class * Recovery time + Distance to community*	638.35	0.73	0.173

Table S3. Local perception of socioeconomic changes following the implementation of Arapaima population management within lakes guarded by any given local community.

Benefits	Brief explanation	Relative importance score (%)
**Income generation**	Fisheries income is accrued by individual households as an annual windfall payment. This substantial amount of money enables several investments that were previously not feasible.	1 (100%)
**Cultural maintenance**	Arapaima fisheries is a deeply entrenched traditional extractive activity. With the establishment of CBM, this activity and traditional knowledge associated with the target species can be learned by adolescents and children, thereby perpetuating this practice.	2 (75%)
**Increasing local “pride”**	Professional fishermen reported an increase in community “pride”. Essentially, arapaima management increases the self-respect, pride and self-esteem of the local community associated with community-based management.	3 (68%)
**Enhanced income distribution**	Prior to CBM, only experienced families could benefit from the arapaima fishery. Following the implementation of management, all community members can participate.	4 (27.5%)

Table S4. Floodplain lake identification, geographic coordinates, and environmental variables associated with each lake. Lake: lake identity; Community: identity of nearest local community; Protected Area: inside or outside a reserve; Man.Class: class of lake management; Lake area: area (ha); Water type: black or white; Perimeter: perimeter of the lake; Dist.Comm.: nonlinear distance on foot to nearest community; Geographic coordinates: Latitude and longitude; Lim.Var.: availability of limnological data obtained in situ during both high and low-water seasons; Pop.Size: arapaima population size (adults and juveniles) based on annual counts

Lake ID	Community	Protect	Man.Class	Lake area	Water type	Perimeter	Dist Comm.	Latitude	Longitude	Lim.Var.	Pop.Size
**Acurau**	Xeruã	reserve	Subsistence	42	White	4750	3736.8	6° 1'52.12"S	67°47'25.28"W	yes	22,000
**Anaxiqui**	Sao José	reserve	Protected	173	White	2085	67.5	5°43'33.34"S	67°48'24.72"W	yes	592,000
**Andreza**	Novo Horizonte	reserve	Unprotected	90	White	5456	2962.5	5° 5'53.35"S	67° 8'25.27"W	yes	4,000
**Angelim**	Nova União	reserve	Subsistence	7	Black	2790	3451.1	5°22'2.00"S	67°27'15.62"W	no	23,000
**Aruanã**	Nova União	reserve	Protected	16	Black	2823	3491.4	5°20'46.07"S	67°25'27.01"W	yes	70,000
**Baliera**	Nova União	reserve	Subsistence	21	White	5317	3406.9	5°23'5.89"S	67°21'28.72"W	yes	24,000
**Bauana**	Bauana	reserve	Unprotected	93.3	White	7000	1054	5°25'45.11"S	67°18'53.43"W	yes	8,000
**Bom Fim**	Bom Fim	reserve	Protected	220	White	1486	686.4	6° 0'19.82"S	67°52'33.92"W	no	345,000
**Boto**	Bom Jesus	reserve	Protected	92	Black	8814	3378.7	5°23'56.20"S	67°14'54.93"W	yes	298,000
**Boto**	Nova União	reserve	Subsistence	11	Black	3492	4580.4	5°19'43.82"S	67°24'56.91"W	no	15,000
**Braga**	Concordia	outside	Protected	25	Black	5602	2242.1	4°41'2.23"S	66°37'28.54"W	yes	176,000
**Branco**	Vista Alegre	outside	Protected	255	Black	9056	3811	5°11'22.19"S	67°16'38.85"W	yes	77,000
**Branco**	Fortuna	reserve	Protected	15	White	2918	2274.2	4°43'3.43"S	66°39'59.17"W	yes	172,000
**Cametá**	Boa Vista	reserve	Subsistence	6	Black	1926	1203.5	4°45'29.79"S	66°43'0.22"W	no	7,000
**Camponesa**	Xibauazinho	reserve	Subsistence	13	Black	3972	3899.5	5°56'15.48"S	67°45'12.77"W	no	52,000
**Canico**	Lago Cerrado	outside	Unprotected	306	White	1717	6540.2	4°47'10.39"S	66°48'30.17"W	yes	0,000
**Cobras**	Monte Carmelo	reserve	Subsistence	4	Black	2213	4511.2	5°24'36.01"S	67°16'57.95"W	yes	26,000
**Comprido**	Roque	reserve	Protected	12	White	3017	3840.4	5° 7'41.20"S	67°12'47.71"W	yes	137,000
**Curape**	Lago Cerrado	outside	UnProtected	573	White	32244	5491	4°42'56.13"S	66°47'26.95"W	yes	22,000
**Damião**	São Francisco	reserve	Subsistence	46	White	3653	1069.7	5°41'12.27"S	67°46'9.55"W	no	38,000
**Deserto**	Santo Antonio	reserve	Subsistence	105	White	20706	4663.6	4°33'34.62"S	66°43'30.51"W	yes	37,000
**Doca**	São Raimundo	reserve	Subsistence	113	Black	7450	5062.9	5°27'51.3"S	67°30'52.3" W	no	63,000
**Dona Maria**	Sao Raimundo	reserve	Protected	101	Black	9926	137.2	5°25'21.48"S	67°31'17.38"W	yes	97,000
**Esperança**	Sao Raimundo	reserve	Protected	108	Black	6711	6507.1	5°25'12.63"S	67°15'26.63"W	no	45,000
**Farias**	Morada Nova	reserve	Subsistence	16	White	1955	3437.7	5°11'16.22"S	67°18'12.81"W	no	29,000
**Florenço**	Morada Nova	reserve	Subsistence	3	Black	865	3173.1	5°29'21.33"S	67°35'49.46"W	no	26,000
**Grande**	Concordia	outside	Protected	88	Black	5681	102.9	4°34'30.06"S	66°37'47.57"W	yes	84,000

**Grande**	Lago Cerrado	outside	Protected	294	White	2048	137.2	4°44'39.55"S	66°42'54.41"W	yes	473,000
**Henrique**	Fortuna	reserve	Subsistence	17	Black	5031	7825.2	5°14'6.40"S	67°18'18.24"W	no	80,000
**Ilha**	Sao Raimundo	reserve	Subsistence	3	Black	1368	4662.6	05°28'35,2"S	67°31'43,3" W	no	67,000
**Istume**	Fortuna	reserve	Subsistence	20	Black	3440	6395.1	5°30'15.13"S	67°36'44.81"W	no	126,000
**Itabaiana**	Xeruã	reserve	Protected	28	Black	6408	5747.1	6° 3'9.73"S	67°45'53.56"W	yes	82,000
**Janiceto**	Morada Nova	reserve	Subsistence	6	Black	2562	2447	5°30'9.20"S	67°36'17.74"W	yes	13,000
**Jiburi**	Fortuna	outside	Unprotected	88.8	White	11000	10010	5°13'7.45"S	67°12'28.67"W	no	6,000
**Limoeiro**	Morada Nova	reserve	Subsistence	28	White	2557	3844.2	5°28'46.44"S	67°34'24.72"W	no	27,000
**Luis Ceará**	Fortuna	reserve	Subsistence	4	Black	1593	7022.3	5°13'36.30"S	67°17'44.90"W	no	23,000
**Macaco**	Xibauazinho	reserve	Protected	53	White	5912	10	5°58'38.99"S	67°46'2.14"W	no	429,000
**Maia**	Caroçal	reserve	Subsistence	15	Black	5187	5736	5°54'14.59"S	67°48'10.04"W	no	10,000
**Mamuria**	Concordia	outside	Unprotected	81	White	5537	4274.4	4°41'55.62"S	66°38'56.30"W	yes	4,000
**Manaria**	Sao Raimundo	reserve	Protected	293	White	2433	121	5°27'58.27"S	67°31'20.15"W	yes	1013,000
**Mandioca**	Mandioca	reserve	Protected	200	White	1372	1443.8	5°52'16.15"S	67°48'19.72"W	yes	482,000
**Mandioquinha**	Mandioca	reserve	Subsistence	129	White	8739	4373.1	5°52'51.23"S	67°49'15.26"W	no	37,000
**Marari Grande**	Xibauazinho	reserve	Protected	269	White	2040	63	5°56'27.74"S	67°45'58.93"W	yes	1509,000
**Maravilha**	Maravilha	outside	Subsistence	368	White	2344	900	6° 6'6.69"S	67°56'1.34"W	yes	71,000
**Marinho**	Concordia	outside	Subsistence	41	White	3396	1000	4°35'1.30"S	66°37'35.14"W	yes	34,000
**Maximiano**	Monte Carmelo	reserve	Unprotected	51.4	White	3700	5039	5°45'40.09"S	67°48'46.91"W	no	5,000
**Mutum**	Vista Alegre	outside	Protected	22	Black	3833	4640.2	4°43'26.29"S	66°40'43.27"W	yes	85,000
**Onças**	Morada Nova	reserve	Subsistence	11	White	2614	1393.6	5°32'33.65"S	67°36'8.30"W	yes	17,000
**Paranã Manariã**	Sao Raimundo	reserve	Protected	132	White	23989.9	3499.6	5°27'9.66"S	67°30'34.76"W	no	290,000
**Patocino**	Xibauazinho	reserve	Subsistence	7	White	2787	5060.7	5°53'09.7"S	67°46'23.2"W	no	54,000
**Pau Furado**	Monte Carmelo	reserve	Subsistence	38	White	2958	4333.9	5°44'39.52"S	67°47'49.44"W	no	22,000
**Pé da Terra**	Xeruã	reserve	Protected	5	Black	2172	8245.1	6° 2'38.32"S	67°44'59.58"W	no	63,000
**Pirapitinga**	Concordia	outside	Protected	6	Black	1546	2823.3	4°40'4.89"S	66°37'30.14"W	yes	94,000
**Ponga**	Concordia	outside	Unprotected	132	White	1102	7200	4°39'26.09"S	66°36'24.08"W	yes	9,000
**Preto**	Nova Esperança	reserve	Protected	54	White	5342	183.9	4°37'10.57"S	66°42'45.20"W	yes	182,000
**Puça**	Xué	reserve	Unprotected	85	White	7233	2495.7	5°35'32.12"S	67°33'40.08"W	yes	0,000
**Pupunha Baixo**	Pupunha	reserve	Unprotected	138.5	White	9929	1500	5°35'36.99"S	67°45'52.18"W	no	13,000

**Raimundão**	Vista Alegre	outside	Protected	14	White	2076	7343.8	4°44'19.73"S	66°40'6.21"W	yes	23,000
**Rato**	Caroçal	reserve	Protected	335	White	2034	50	5°43'4.05"S	67°43'45.11"W	yes	663,000
**Recreio**	São Raimundo	reserve	Subsistence	86	White	4532	3246.7	5°25'56.62"S	67°32'31.35"W	no	16,000
**Redondo**	Xibauazinho	reserve	Subsistence	30	White	2688	1378.2	5°59'04.0"S	67°46'57.5" W	no	47,000
**Redondo**	Novo Horizonte	reserve	Unprotected	82.4	White	3310.9	1047	5° 4'9.08"S	67° 7'43.65"W	no	9,000
**Ressaca Xibaua**	Xibauá	reserve	Subsistence	143	Black	1132	4375.4	5°53'44.64"S	67°54'7.67"W	no	18,000
**Roque**	Roque	reserve	Unprotected	90.8	White	8710	1500	5° 6'18.36"S	67°12'4.41"W	no	4,000
**Sacado Eré**	Goiabal	outside	Unprotected	306	White	16926.3	3590	5° 7'3.77"S	66°59'27.15"W	no	18,000
**Sacado Jiburi**	Fortuna	reserve	Protected	412	White	2879	57	5° 8'48.67"S	67°13'29.31"W	yes	627,000
**Sacado Mari**	São Raimundo	reserve	Protected	282	White	1730	34	5°25'11.63"S	67°26'47.90"W	no	87,000
**Samauma**	Morada Nova	reserve	Protected	105	White	8747	3302.8	5°31'39.36"S	67°38'4.09"W	yes	643,000
**Santa Clara**	Xibauazinho	reserve	Unprotected	200	White	1452	1918.2	5°57'59.40"S	67°49'45.58"W	yes	7,000
**Santa Cruz**	Xibauazinho	reserve	Subsistence	8	White	2963	7090.9	5° 51'43.1"S	67° 45'70.8"W	no	42,000
**Santa Fé**	Concordia	outside	Unprotected	401	White	2766	2700	4°38'18.60"S	66°38'17.61"W	yes	3,000
**Santo Antonio**	Santo Antonio	reserve	Unprotected	53	White	4664	2443.8	5°33'9.06"S	67°33'33.43"W	yes	5,000
**São Sebastião**	São Sebastião	outside	Unprotected	344	White	2375	500	6° 3'33.20"S	67°52'39.11"W	yes	16,000
**Socó**	São Raimundo	reserve	Subsistence	10	White	1683	5950.6	05°22'41.6"S	67°30'27.1"W	no	11,000
**Tambaqui**	São Raimundo	reserve	Subsistence	7	White	2037	6820.6	4°32'43.35"S	66°40'3.96"W	no	23,000
**Tangara**	Monte Carmelo	reserve	Unprotected	37	Black	9098	5111.3	5°45'12.14"S	67°43'59.50"W	yes	31,000
**Toaré**	Xeruã	reserve	Protected	9	Black	1989	6767.4	6° 2'8.93"S	67°46'23.90"W	yes	43,000
**Torcate**	Monte Carmelo	reserve	Protected	108	Black	4783	1850	5°43'50.77"S	67°46'34.38"W	yes	85,000
**Tracaja**	Morada Nova	reserve	Protected	14	Black	2069	4045.6	5°52'47.34"S	67°51'5.74"W	no	66,000
**Tucunare**	Ouro Preto	reserve	Subsistence	6	Black	1654	8966.4	5°14'38.33"S	67°18'7.59"W	no	20,000
**Veado**	Toari	reserve	Protected	183	White	1940	100	5°49'36.85"S	67°47'55.27"W	no	418,000
**Xibauá**	Xibauá	reserve	Subsistence	17	Black	2023	1590.2	5°52'59.30"S	67°53'5.85"W	no	28,000
**Zé**	Xibauazinho	reserve	Subsistence	5	Black	2200	7234.7	6°00'51.1"S	67°52'14.3"W	no	67,000

2. Supplementary Information Figures

Figure S1. Adult arapaima being weighed by a local extended family of arapaima managers, following a capture from a managed (protected) oxbow lake.

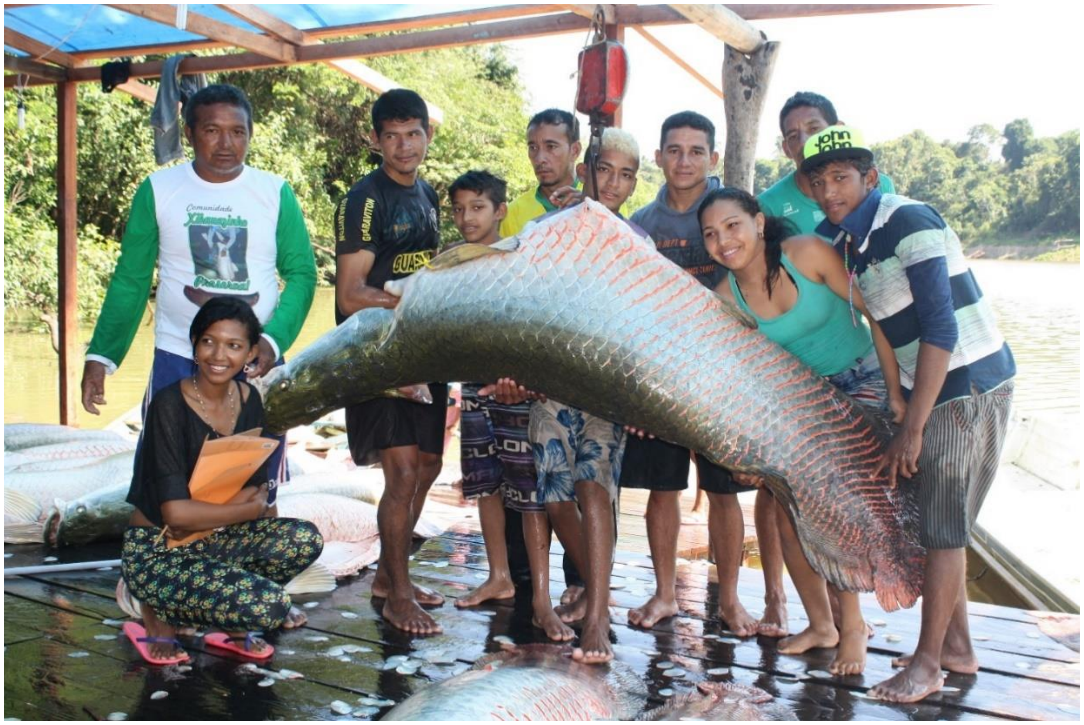

Figure S2. Arapaima population size in relation to floodplain lake management class and protected area context. Green and red boxes (showing median values, lower and upper quartiles and outliers), represent lakes located either inside or outside protected areas, respectively.

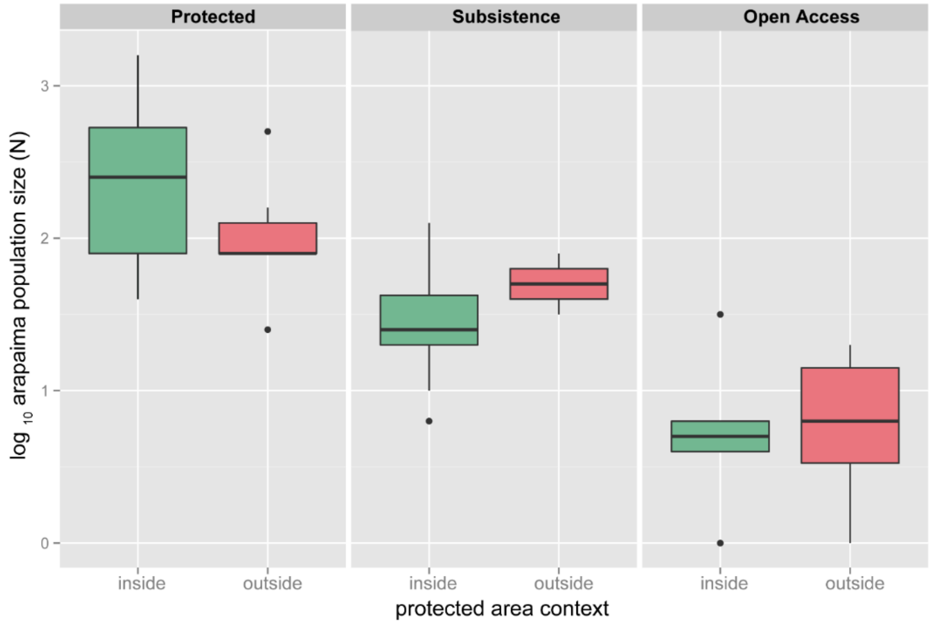

Figure S3 . Arapaima population sizes for both adults (grey boxes) and juveniles (yellow boxes) across the three classes of floodplain lakes in terms of implementation of community-based fisheries management. Box plots show median values, lower and upper quartiles, and outliers.

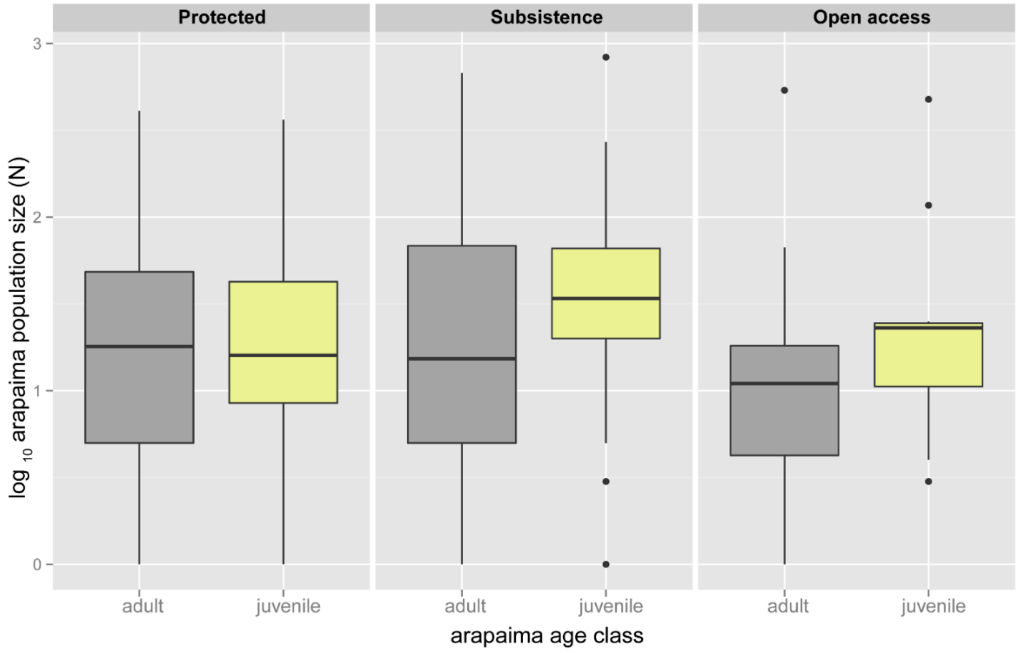

Figure S4. Arapaima population recovery trajectories over time for individual floodplain lakes for both (A) adults and (B) juveniles (see text). Individual lines represent annual time series for any given lake for which data from repeated multi-year annual counts were available. Blue lines indicate protected and subsistence lakes; red lines indicate open-access lakes.

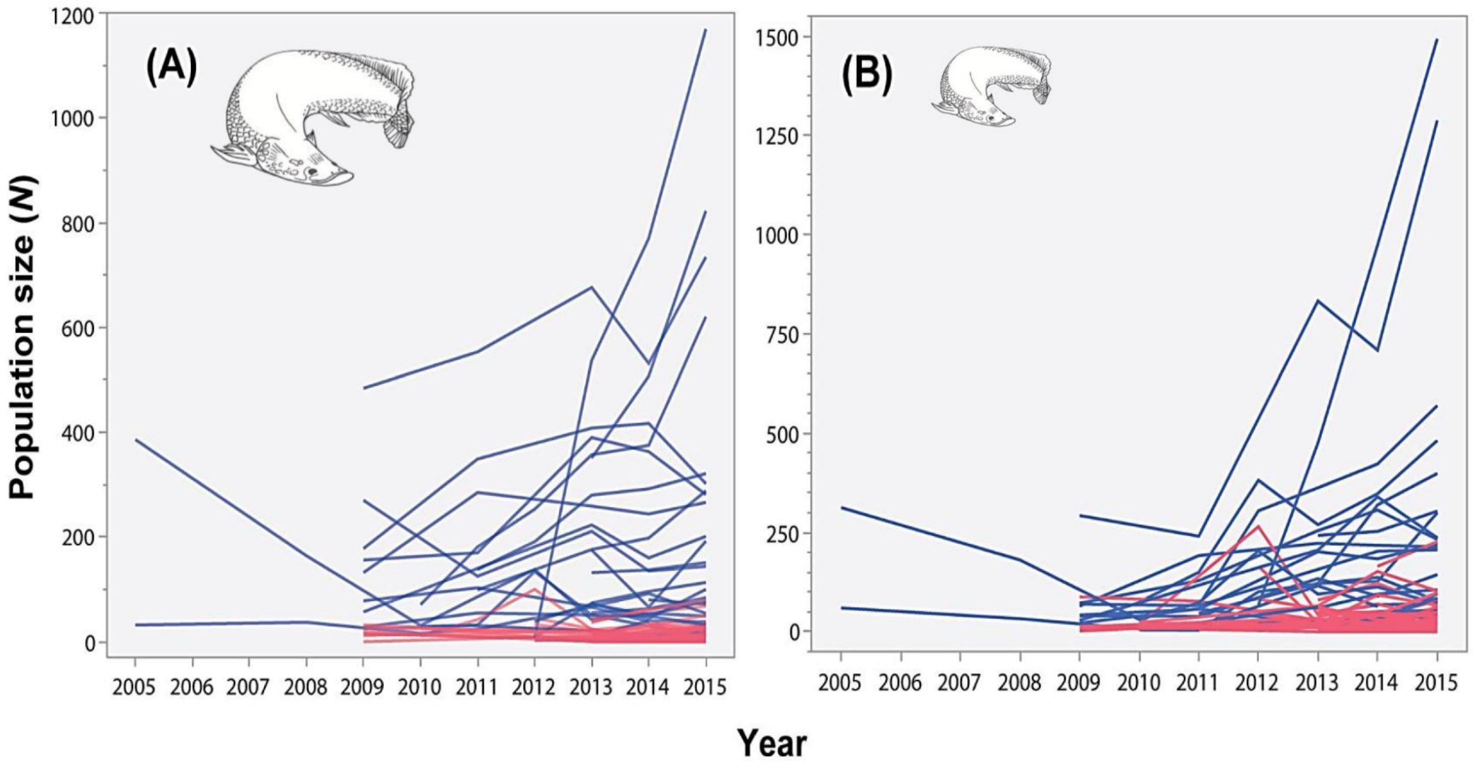

Figure S5. Arapaima population size (based on annual counts) within floodplain lakes as a function of physical nonlinear distance travelled (sqrt x) on foot from the nearest local community.

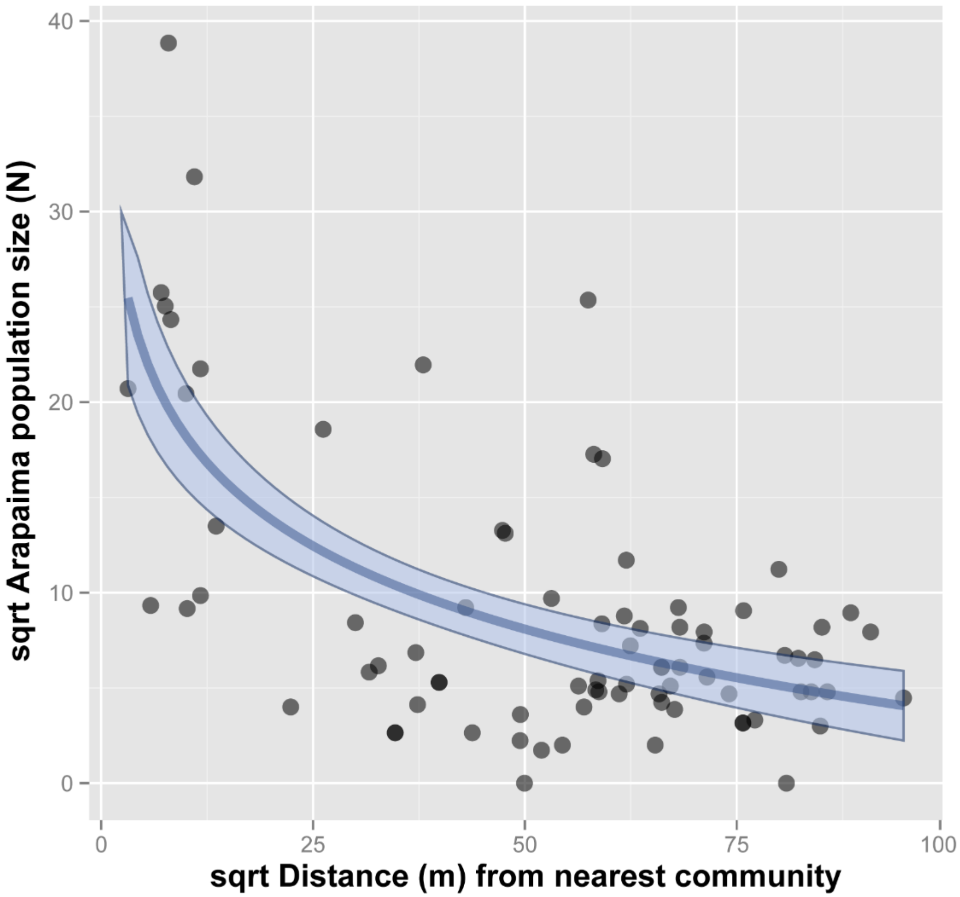

Figure S6. Model predictions of the time lag (yrs) required to achieve a target population recovery of a local arapaima population size of 1000 individuals, given the rates of

population growth observed in either white-water (blue curve) or black-water protected lakes (red curve), managed by local communities. Arrows indicate 95% confidence intervals.

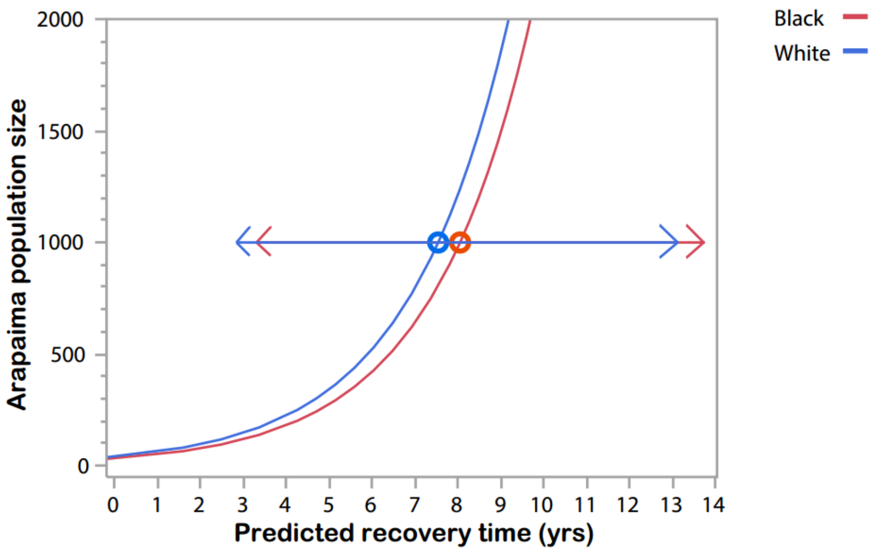

1. Supplementary Information Video

Video 1. Systematic annual counts of local arapaima populations within community-managed and unmanaged oxbow lakes, which take place along the Juruá River of Western Brazilian Amazonia, are largely based on the surfacing events of this air-breathing fish. This short video shows the exact moment of a breathing event during which an adult arapaima comes to the surface. On the basis of the water commotion generated by these events, experienced local fishermen can also distinguish two main size classes (adults and juveniles).
